# Midwives

**Published:** 1886-01

**Authors:** 


### Midwives.

There are, in Erie county, about 100 women who have been practicing midwifery. Of these, forty-eight have, under the new law, presented themselves for examination. We publish herewith a complete list of the names of those who have obtained a license to practice from the Board of Examiners:

| | |
|---|---|
| Bartel, Eva | Lachine, Anna |
| Bischoff, Amelia | Levy, Mary |
| Brown, Francis | Looney, Honora |
| Dory, Barbara | Madel, Francisca |
| Feinen, Susanna | Maschke, Mena |
| Freer, Ellen | Menrich, Rosina |
| Fretschi, Maria | Miller, Joanna |
| Gau, Julia | Miller, Renatia |
| Grindel, Maria | Miller, Teresa |
| Haffa, Christine | Owecka, Veronika |
| Hildenbrand, Louise | Riedell, Bertha |
| Hoffmann, Elizabeth | Shabio, Charlotte |
| Janner, Caroline | Stoehr, Anna |
| Kane, Bridget | Urtel, Emilia |
| Kleinhaus, Rufina | Wagner, Augusta |
| Klock, Anna | Wienert, Mary |
| Kurtzman, Anna | Wolfe, Margaret |

